# Infected chronic ischemic wound topically treated with a multi-strain probiotic formulation: a novel tailored treatment strategy

**DOI:** 10.1186/s12967-019-2111-0

**Published:** 2019-11-09

**Authors:** Salvatore Venosi, Giancarlo Ceccarelli, Massimiliano de Angelis, Luca Laghi, Laura Bianchi, Ombretta Martinelli, Debora Maruca, Eugenio Nelson Cavallari, Fabrizia Toscanella, Paolo Vassalini, Vito Trinchieri, Alessandra Oliva, Gabriella d’Ettorre

**Affiliations:** 1 Department of Cardio-Thoraco-Vascular, Surgery and Transplants, University of Rome Sapienza, Rome, Italy; 2 Department of Public Health and Infectious Diseases, University of Rome Sapienza, Viale del Policlinico 155, Rome, Italy; 3 Department of Agri-Food Science and Technology, University of Bologna, Bologna, Italy; 4 Functional Proteomic Laboratory, Department of Life Sciences, University of Siena, Siena, Italy; 5 Diabetic Foot Center, Istituto Nazionale Ricovero e Cura Anziani (INRCA), Ancona, Italy

**Keywords:** Bacteriotherapy, Topical probiotic, Metabolomic, Wound, Wound care, Wound healing, Antimicrobial resistance

## Abstract

**Background:**

A wide debate is ongoing regarding the role of cutaneous dysbiosis in the pathogenesis and evolution of difficult-to-treat chronic wounds. Nowadays, probiotic treatment considered as an useful tool to counteract dysbiosis but the evidence in regard to their therapeutic use in the setting of difficult-to-treat cutaneous ulcers is still poor.

**Aim: clinical report:**

An 83-year-old woman suffering a critical limb ischemia and an infected difficult-to-treat ulcerated cutaneous lesion of the right leg, was complementary treated with local application of a mixture of probiotic bacteria.

**Methods:**

Microbiological and metabolomic analysis were conducted on wound swabs obtained before and after bacteriotherapy.

**Results:**

During the treatment course, a progressive healing of the lesion was observed with microbiological resolution of the polymicrobial infection of the wound. Metabolomic analysis showed a significant difference in the local concentration of propionate, 2-hydroxyisovalerate, 2-oxoisocaproate, 2,3-butanediol, putrescine, thymine, and trimethylamine before and after bacteriotherapy.

**Conclusion:**

The microbiological and metabolomic results seem to confirm the usefulness of complementary probiotic treatment in difficult-to-treat infected wounds. Further investigations are needed to confirm these preliminary findings.

## Background

Wound healing is a multi-layered process consisting of sequential overlapping phases, involving soluble mediators, blood and parenchymal cells [1]. This process is intrinsically linked to immune cells response and inflammation. In recent years, new insights in the pathogenesis of chronic wounds highlighted the importance of cutaneous microbiome and skin dysbiosis. Polymicrobial biofilm, which foster pathogenic microbial growth and disrupts the coordinated events involved wound healing, are highly abundant in chronic wounds and play a crucial role in the pathogenesis of impaired cutaneous healing [2]. In addition, polymicrobial biofilms can impair the efficacy of antibiotic therapy, thus leading to its overuse and to the subsequent development of antimicrobial resistance (AMR). Certain probiotics have been demonstrated to be active against pathogens through competition for survival resources as well as the production of organic acids and antimicrobial substances [3]. Therefore, theoretically probiotics might represent a powerful strategy to promote wound healing in subjects with skin lesions colonized or infected by multi drug resistant bacteria (MDR). Herein, we report a compassionate case of an 83 year old woman successfully treated with topical administration of probiotics for an ischemic and infected (polymicrobial infection: *Klebsiella pneumonias, Enterococcus faecalis* and *Proteus mirabilis*) chronic wound.

## Clinical case

A 83-year-old woman affected by Critical Limb Ischemia (CLI) developed an ulcerated cutaneous lesion of the right leg, extended to the second and third finger of the homolateral foot. Her medical history showed type II diabetes mellitus, systemic arterial hypertension, ischemic heart disease (previous coronary artery bypass surgery) and atrial fibrillation; she was also an active cigarette smoker (20 cigarettes/day for 40 years). On physical examination, the above mentioned cutaneous ulceration showed anterior and posterior necrotic eschars; moreover, acupressure provoked a lively tenderness and pain inhibited leg motility. Finally, bilateral femoral pulses were present while distal peripheral pulses were absent (popliteal, posterior tibial and pedidial artery). Ankle Brachial Index (ABI) was bilaterally not assessable.

On admission, blood exams showed C-reactive protein (CRP) 101,000 μg/L, erythrocyte sedimentation rate (ESR) 100 mm/h, hemoglobin (HGB) 8.0 g/dl, platelets count 454,000 μg/L, white blood cells count (WBC) 12,800/μl, international normalized ratio (INR) 1.87, partial thromboplastin time (PTT) ratio 2.3. Surgical management consisted of recanalization and percutaneous transluminal angioplasty **(**PTA) with drug eluting balloon (DEB) ranger 5 × 100 mm in superficial femoral artery (SFA) and right popliteal artery, followed by surgical curettage of necrotic forefoot injuries and amputation of the second toe of the right foot. The infectious diseases specialist recommended to start systemic antibiotic therapy with piperacillin/tazobactam 4.5 g I.V. every 8 h.

After 8 days, an improvement of inflammatory markers was observed, and antibiotic therapy was switched to oral minocycline100 mg (1 tablet every 12 h) for 15 days. The patient was discharged after a total of 21 days of hospitalization. At home, the patient underwent local dressing of wound and amputation margins with antiseptic compress solution twice a week after cleansing, furthermore a polymeric membrane (PolyMem^®^-Ferries Mfg) was routinely applied.

36 days after hospital discharge, given the worsening of local condition with considerable increase in exudation, despite a 4 weeks course of antibiotic therapy with Amoxicillin (500 mg orally every 12 h) empirically prescribed by the treating physician, the patient referred to our University Center. At the time of first observation, the wound appeared moist with abundant secretion and covered with fibrin. A wound swab was routinely performed and confirmed the previous evidence of positivity for MDR *K. pneumoniae*, *E. faecalis* and *P. mirabilis*. Although the patient was afebrile, three blood cultures, that tested negative, were performed.

Give the multiple microorganisms present on the wound and the severely prostrated general conditions of the patient, it was decided to start medications with 10% cutaneous-iodopovidone solution for topical use (Poviderm^®^ 10% Skin Solution). After 30 days, since no improvement of the wound was observed, systemic antibiotic treatment was suspended. In reason of the previous therapeutic failures, the possibility of treating the wound with a mixture of probiotics as a rescue therapy was considered; written informed consent was obtained and subsequently a mixture of probiotic bacteria (lyophilized powder sachets, each containing 100 billion colony forming unit (CFU) of *Lactobacillus plantarum* NCIBMB 43029 20% in weight, *Lactobacillus acidophilus* NCIBMB 43030 20% in weight and *Streptococcus thermophilus* NCIMB 30438 40% in weight) (provided by Mendes SA, Lugano, Switzerland) was topically applied three times/week. Probiotic treatment was provided by Mendes SA, Lugano, Switzerland.

**Fig. 1:**
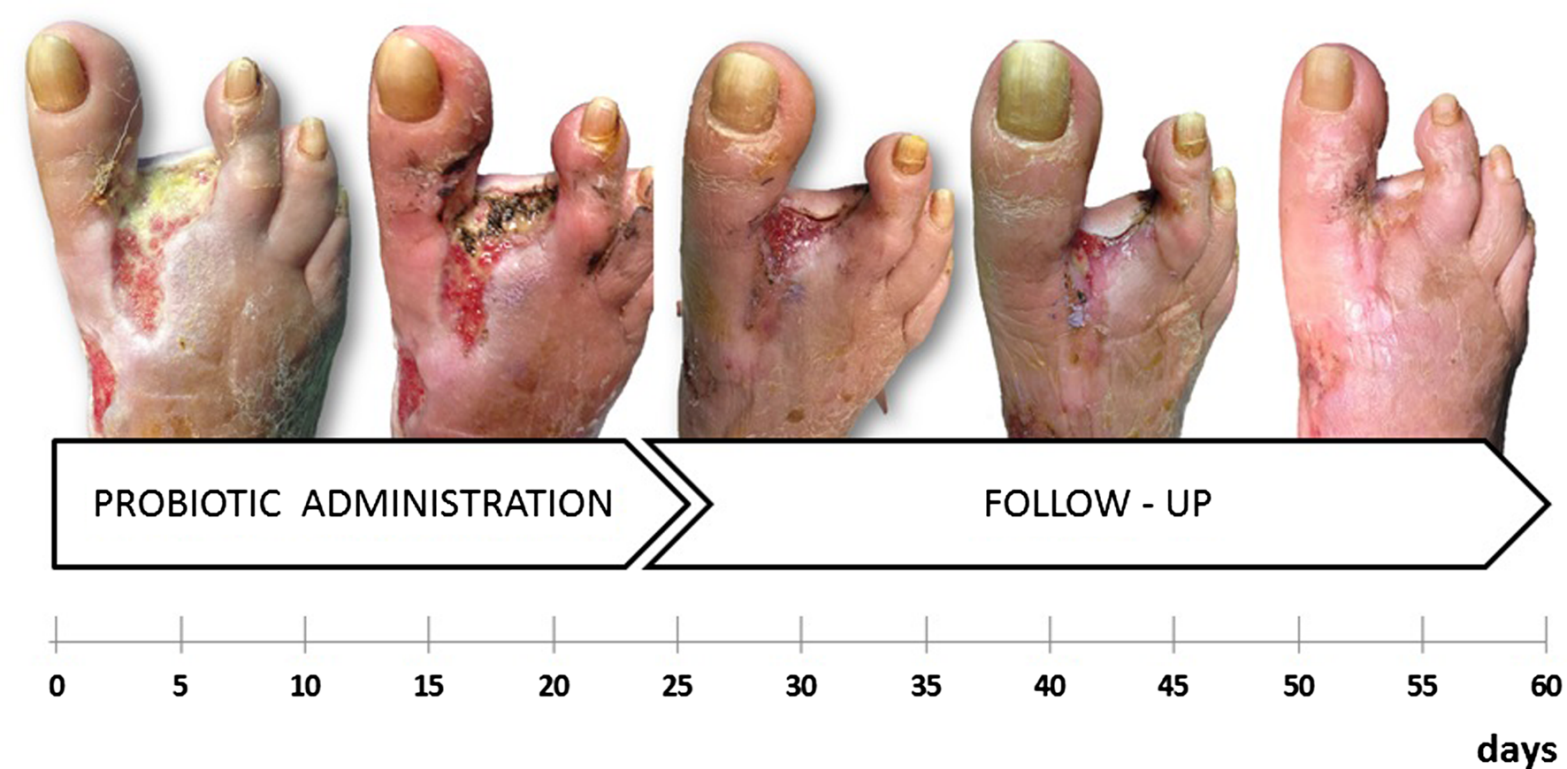
Dynamics of clinical changes and healing of infected wound

The conditions of the wound appeared stable after 1 week of probiotic treatment. Following 2 weeks of treatment a modification in microbiological results and a slowly progressive healing of the lesion were noted. The wound pad tested negative for *E. faecalis* at day 48 (12 days after the start of the probiotic treatment) and negative for *K. pneumoniae* and *P. mirabilis* at day 57 (after 21 days of the topical probiotic treatment). In the initial phase of treatment, an entire probiotic sachet (100 billion CFU) was applied three times/week, while in subsequent period the applied amount was proportional to the residual size of the wound. Systemic or topical antibiotic treatment was not administered during the course of probiotics. The overall duration of probiotic application was 24 days (Fig. 1).

During the subsequent 90 days, a slow but complete wound healing was observed; PolyMem dressings were applied by the patient to improve comfort in reason of the fact that autonomous walking and other daily tasks were resumed. Furthermore, normalization of serum inflammatory markers (erythrocyte sedimentation rate (ESR) and CRP) was observed.

## Materials and methods

Wound swabs, collected before and after bacteriotherapy, were examined.

### Microbiological analyses

Swabs were collected according to the available guidelines, before probiotic administration and after 12, 21 and 24 days of probiotic therapy [4]. After collection, wound swabs were plated on both aerobic and anaerobic plates and incubated at 37 °C for 24 h. Bacterial identification and antimicrobial susceptibility tests were performed with VITEK-2 system (bioMérieux, Italia Spa).

Until further analyses, bacteria were stored on cryovial bead preservation system (Microbank; Pro-Lab Diagnostics, Richmond Hill, ON, Canada) at − 80 °C; the inoculum was prepared by spreading one cryovial bead on blood agar plate and incubating overnight at 37 °C. One colony was resuspended in 3 ml of NaCl and then adjusted to a turbidity of 0.5 McFarland, corresponding to ≈ 1.5 × 10^8^ CFU/ml.

Antimicrobial activity of the probiotic mix was tested on *K. pneumoniae*, *P. mirabilis* and *E. faecalis*. Briefly, probiotic formulations were dissolved in 10 ml of M.R.S. broth. After 30 min of incubation at room temperature, 450 µl of the obtained solution were added to 450 µl of M.R.S. broth with a final volume of 900 µl (1:2 dilution). A final bacterial inoculum of 5 × 10^5^ CFU/ml was added to tubes containing twofold serial dilutions (1:2 to 1:256) of M.R.S. broth plus probiotic and incubated at 37 °C for 24 h. Minimal bactericidal dilution (MBD) was defined as the lowest dilution that induced a ≥ 99.9% (i.e., ≥ 3-log10 CFU/ml) reduction of the initial bacterial count after 24 h of incubation.

### Metabolomics

For metabolomic investigation, 250 mg of PolyMem sponges with absorbed exudates was added to 1 ml of bidistilled water and vortex mixed for 5 min. After centrifugation at 18,630×*g* for 15 min at 4 °C, 0.7 ml of supernatant were added to 0.1 ml of a D_2_O solution of 3-(trimethylsilyl)-propionic-2,2,3,3-d4 acid (TSP) sodium salt 10 mmol/L, buffered at pH 7.00 ± 0.02 by means of 1 M phosphate buffer, containing also NaN_3_ 2 mmol/l. TSP was employed as an Nuclear Magnetic Resonance (NMR) chemical-shift reference, while NaN_3_ avoided microbial proliferation. After a second centrifugation at the above conditions, ^1^H-NMR spectra were recorded at 298 K with an AVANCE III spectrometer (Bruker, Milan, Italy), at a frequency of 600.13 MHz, equipped with Topspin software (Ver. 3.5). Following Zhu et al. [5], the signals from broad resonances were suppressed by a CPMG-filter composed by 400 echoes with an echo time of 0.4 ms and a 180° pulse of 0.024 ms, for a total filter of 330 ms. The deuterium oxide residual signal was suppressed by means of pre-saturation. Topspin software was used to adjust the phase of each spectrum, while R computational language (R Development Core Team) was employed for any further spectra processing, molecules quantification and data mining step, by means of scripts developed in house as detailed elsewhere [6, 7]. Signals assignment was performed by comparing chemical shift and multiplicity with the Human Metabolome Database and the compounds library (Ver. 10) of Chenomx software (Chenomx Inc., Canada, Ver. 8.3) [8].

After identification and quantification, molecules underwent pathway enrichment analysis, by querying KEGG biochemical pathways database (https://www.genome.jp) through MetaboAnalyst (ver. 4.0) web interface (https://www.metaboanalyst.ca) [9].

## Results

### Microbiological analyses

Prior to probiotic therapy, wound swab grew *K. pneumoniae*, *P. mirabilis* and *E. faecalis* whereas at day 12 only *K. pneumoniae* and *P. mirabilis* showed growth and at day 21 the wound swab did not show bacterial growth. Absence of bacterial growth was confirmed also at day 24 after probiotic therapy. The in vitro MBD of the probiotic formulation against *Klebsiella pneumoniae*, *Enterococcus faecalis* and *Proteus mirabilis*, was 1:16, 1:16 and 1:64, respectively.

### Metabolomics

**Fig. 2:**
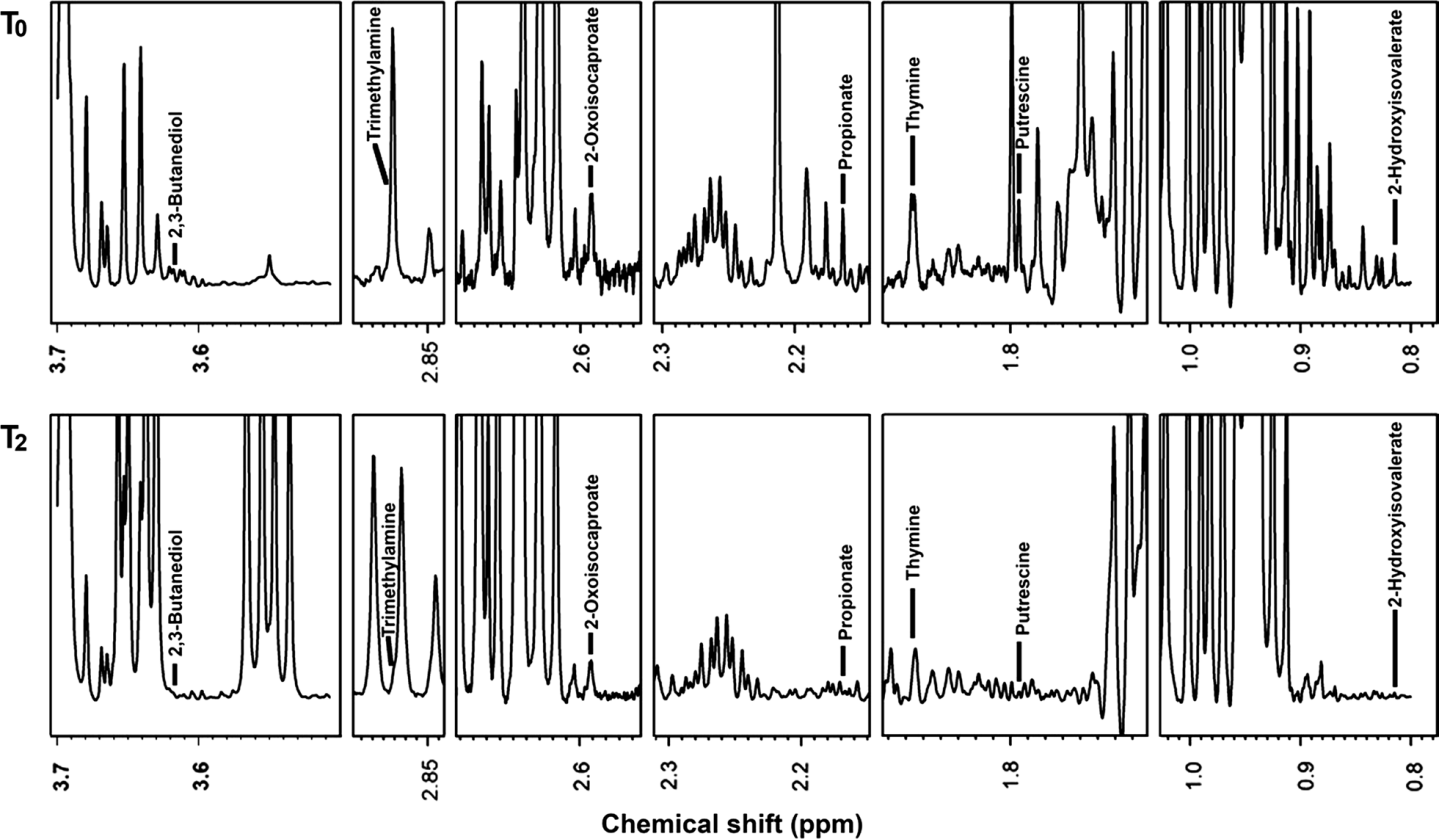
Metabolomics investigation: ^1^H-NMR spectra of molecules obtained by wound swabs before (T0) and after (T2) probiotic administration

**Fig. 3:**
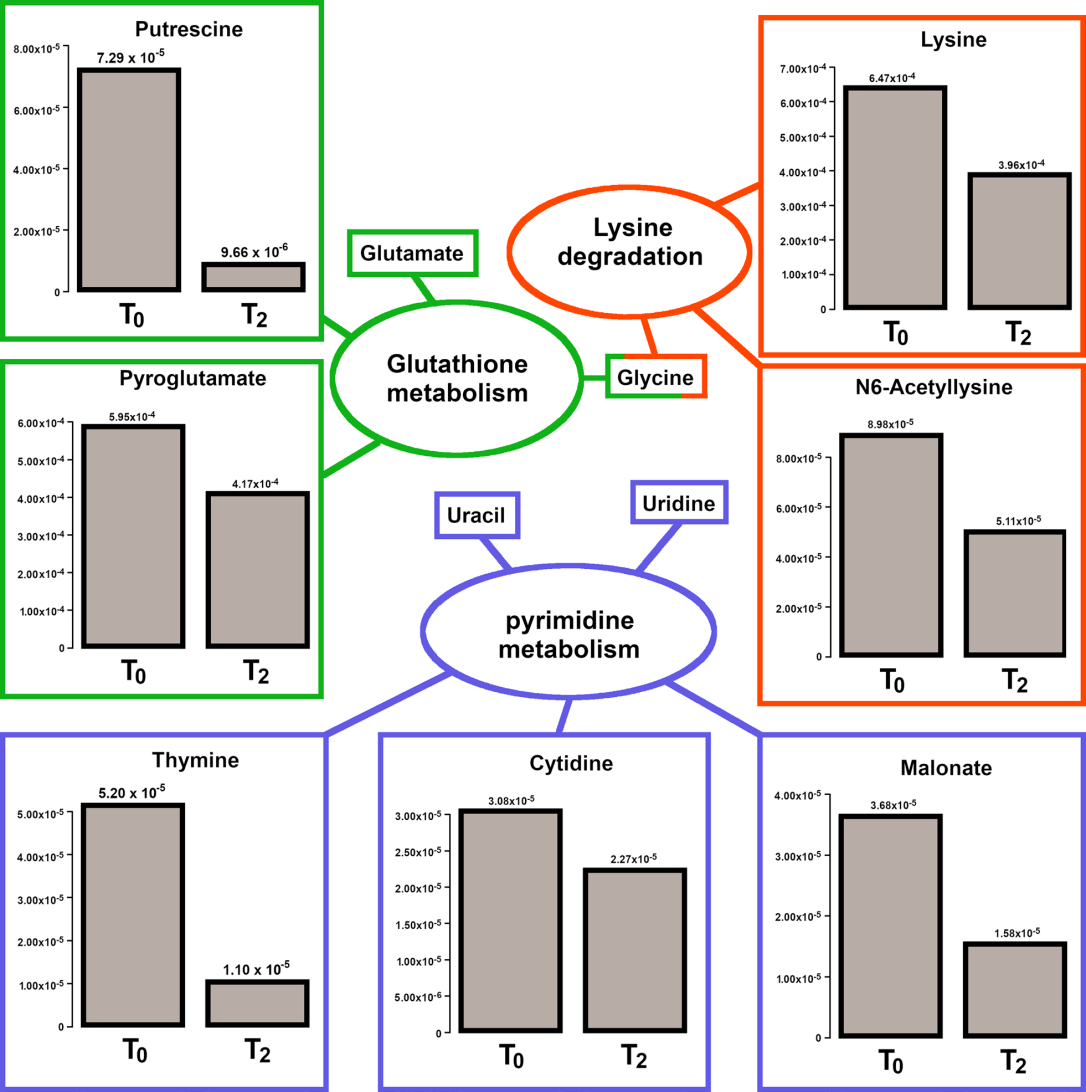
Pathway enrichment analysis on all the molecules quantified by ^1^H-NMR. The barplots report the concentrations of the molecules differing by more than 30% in connection to the treatment. Names without barplots report the observed molecules included in the same pathway, differing by less than 30%

Overall, 63 molecules were identified: seventeen of them showed a change in their initial concentration equal or greater than 30% after the treatment with probiotic. Proprionate, 2-hydroxyisovalerate, 2-oxoisocaproate, 2,3-butanediol, putrescine, thymine, and trimethylamine showed the greatest differences in concentration between the time when the wound was infected and the time when it became sterile (Figs. 2 and 3).

## Discussion

Chronic wounds represent a “silent epidemic” that affects a large fraction of the world population [5]. In developed countries, 1–2% of the population is estimated to experience a chronic wound during its lifetime [10]. Despite the fact that a variety of therapeutic modalities and multidisciplinary approaches have been developed, antimicrobial treatments are often ineffective and impaired wound healing remains a major challenge for both health professionals and patients.

Wound healing is a natural biological multi-layered process that can be affected by many factors, among which the interaction of the wound with the skin microflora is one of the most important [11]. At present, 19 bacterial phyla and over 1000 bacterial species have been identified within the most superficial level of skin, including in wounds [12, 13]. Systemic or topic antimicrobial treatments may reduce beneficial bacterial populations and contribute to the emergence of an antibiotic resistant flora, with detrimental effects on wound healing [14, 15].

In about 60% of chronic wounds [2], bacterial biofilm is associated with impaired wound healing. Biofilm is constituted by bacteria embedded in an exo-polymeric substance consisting of polysaccharides, lipids, and proteins [16]. This extracellular matrix acts as a physical barrier that protects the embedded bacteria from antibiotics as well as host defense mechanisms. At the same time, since space and resources are not unlimited, different bacterial species are forced to develop the capability to counteract proliferation of other microorganisms by secreting toxic metabolites or by interfering with competitors’ quorum-sensing [17–20]. This concept, known as ‘bacterial interference’, has been transposed to practice by administering alpha streptococci with the intent to reduce the recurrences of acute and secretory otitis media in children [21].

Probiotics are defined by the World Health Organization as “live microorganisms which, when administered in adequate amounts, confer a health benefit to the host” [22]. Bacteria with probiotic properties have been already reported to be beneficial in the management of diabetes, respiratory tract infections, gastro-intestinal disorders, and uro-genital infections [23–29]. Oral ingestion of probiotics has also been reported to be advantageous in skin inflammatory diseases and chronic wounds [30].

To the knowledge of the authors, this is the first time that a probiotic formulation was applied topically on a chronic ischemic wound infected by three MDR bacteria.

Obviously, the probiotic treatment might exert its positive effects on wound healing not only by counteracting pathogenic bacteria proliferation as evidenced by the results of the in vitro MBD against *K. pneumoniae*, *E. faecalis* and *P. mirabilis*, but also through modulation of host immune response (*e.g.* by inducing cytokine production: tumor necrosis factor (TNF)-α, interferon (IFN)-γ, interleukin (IL)-4, IL-6, transforming growth factor (TGF)-β and matrix metalloproteinases (MMPs) [31]) and by stimulating keratinocyte proliferation and migration [32, 33].

Chronic wounds produce exudates containing high levels of proteases, *i.e.* metalloproteases and elastase, as well as reduced concentrations of tissue protease inhibitors [34]. The unbalanced activity of proteases and their inhibitors leads to chronic degradation of the extracellular matrix with consequent release of “waste amino acid products”, which can be identified by metabolomic analysis. In our patient, the ^1^H-NMR delineated that metabolic pattern of wound exudate drastically changed after the topical probiotic treatment. Seventeen molecules, out of the 63 overall identified, showed a change in their concentration of at least 30% after the treatment (Figs. 2 and 3). In particular, proprionate, 2-hydroxyisovalerate, 2-oxoisocaproate, 2,3-butanediol, putrescine, thymine, and trimethylamine significantly differed between the infected phase and sterile phase of the wound. Pathway enrichment analysis based on the molecules identified and quantified, suggested that three pathways were differently expressed before and after the treatment (Fig. 3). By means of ^1^H-NMR, we could quantify 5 molecules pertaining to the pyrimidine metabolism and the concentration of three of them, namely cytidine, thymine and malonate, was found to decrease by more than 30% in connection to the treatment. A similar decrease characterized also lysine and N6-acetyllysine, two of the three molecules involved in lysine degradation that we were able to quantify. Finally, a decrease of more than 30% characterized also pyroglutamate and putrescine, two of the four molecules involved in glutathione metabolism that we could quantify. The differential expression of each of the three pathways seems to be connected to the modification in bacterial activity in connection to the resolution of infection.

Since lysine is an essential amino acid, the observed decreases of its free form in the wound exudate, which is not regulated endogenously, can be safely considered as a direct consequence of the reduced proteolysis activity locally exerted by bacteria, caused by the resolution of *Proteus* infection.

Circulating pyrimidine nucleobases are tightly regulated by effective cells’ uptake mechanisms [35], in reason of this observation the lower concentration of cytidine and thymine upon infection resolution may reflect the restore of normal functionality of the cells surrounding the wound.

In addition to the three pathways identified, other metabolites quantified by ^1^H-NMR may be functionally related to pathogens proliferating in the infected wound of the reported case.

Liu et al. demonstrated that l-serine, l-valine and l-leucine promote macrophage phagocytosis in *K. pneumoniae* lung infection, thus declining *Klebsiella* concentration and increasing host survival [36]. The high concentration of, bona fide, microbial related metabolites from branched chain amino acids (e.g. propionate [37]) in the infected wound as well as their conspicuous depletion in the probiotic-treated and pathogen-eradicated healed-wound may indeed reflect infection resolution and reduced systemic stress. Moreover, a high reduction of 2-hydroxyisovalerate and 2-hydroxyisocaproate concentrations after the ulcer treatment with probiotics was also detected. These molecules are hydroxyderivate of the alpha-ketoacid catabolites respectively produced by transamination of valine and leucine [38, 39]. Valine and leucine catabolism may proceed toward propionate synthesis or may shift to 2-hydroxyisovalerate and 2-hydroxyisocaproate production. Marked increase in concentrations of branched-chain amino and 2-oxo acids may have toxic effects for the host, as systemically observed in maple syrup urine disease (MSUD) [40].

In the reported case, the massive decrease of 2,3-butenediol in the second wound exudate collected may reflect the clearance of *Klebsiella* infection, as confirmed by microbiological analyses. *K. pneumoniae*, which is one of the most studied microorganisms for the large-scale production of this compound, has in fact an incredible ability to metabolize various substrates to produce high amounts of 2,3-butenediol [41]. A further possible involvement of *Klebsiella* in conditioning the metabolomic pattern we depicted in the infected wound is suggested by the complementary concentration trend of glycerol, formate and succinate. *K. pneumoniae* is commonly used and observed in glycerol fermentation under mesophilic conditions [42]. According to its humectant properties, skin endogenous glycerol, which is produced in pilosebaceous units by triglycerides catabolism, plays a key role in skin hydration maintenance [43]. *Klebsiella* may indeed use skin glycerol as energy source during skin wound infections. According to our data, eradicated *Klebsiella* infection is associated: (i) to glycerol detection, which was not obtained in infected exudate, and (ii) to a significant decrease of fumarate and succinate, both obtained by glycerol fermentation.

Polyamine putrescine is another product normally derived by amino acid that appeared drastically reduced after probiotic treatment. Putrescine plays a role in several steps of bacterial growth and virulence. It is actually involved in biofilm formation, protection from oxidative and acid stress and escape from phagolysosomes [44–46]. Dead tissues are an ideal medium for anaerobic bacteria such as *Bacteroides fragilis*, *Bacteroides prevotella*, *Clostridium perfrigens* and *Fusobacterium nucleatum*, which produce putrescine and cadaverine. Other bacteria, such as *Klebsiella* and *Proteus*, colonize these tissues and contribute to wound smell [47]. Putrescine, which is produced from arginine by the combined activity of SpeA and SpeB proteins, has been reported to be a cell wall component in *P. mirabilis*, in which it is also known to play a crucial role in control of gene expression, swarming, and tissue colonization [48]. Most likely, the high presence of putrescine in the first exudate sample of our patient was related to the presence of a heterogeneous bacterial community, including both anaerobic and aerobic microorganisms even though the drastic reduction of putrescine and propionate in the second exudate is apparently imputed to the ongoing resolution of *Proteus* infection.

The main limitation for this study is that it is a case report and therefore the results presented should be considered with extreme caution. Furthermore, a number of studies reported a variability in both microbial counts and isolates obtained from different parts of chronic wounds. In our case, the culture data (obtained from the center and the edge of the wound sampling) may have been influenced by the combination of these locations within one single swab. Finally, the data obtained with the probiotic mixture used for this patient are not automatically extendable to other probiotics and further studies are needed to better define the potential of the products used.

## Conclusion

To summarize, the probiotic treatment resulted effective against the three bacteria species *K. pneumonia*, *P. mirabilis* and *E. faecalis*, which are notoriously difficult to eradicate from infected wounds. The metabolites identified by ^1^H-NMR in the infected wound exudate were considered to be consequent to the lysis of wound dead tissue and the formation of the exudate itself, which is at least in part determined by the activity of aerobic and anaerobic bacteria. The modifications of certain metabolites were interpreted in the light of a drastic reduction in the bacterial load (as evidenced by clinical microbiological analysis) as well as a consequence of generalized improvement of the wound.

Based on the promising results here discussed, clinical trials should be conducted to confirm the benefits of topical probiotic treatment for the management of chronic wound healing.

## Supplementary information

**Additional file 1: Figure S1.** Molecules which concentration in the wound exudate was found to differ by more than 30% in connection to the treatment.

## Data Availability

Scientific material is available from the authors, upon request.

## Abbreviations

AMR: antimicrobial resistance
MDR: drug resistant bacteria
CLI: Critical Limb Ischemia
ABI: Ankle Brachial Index
CRP: C-reactive protein
ESR: erythrocyte sedimentation rate
HGB: haemoglobin
WBC: white blood cells count
INR: international normalized ratio
PTT: partial thromboplastin time
PTA: percutaneous transluminal angioplasty
DEB: drug eluting balloon
SFA: superficial femoral artery
*K. pneumoniae*: *Klebsiella pneumoniae*
*E. faecalis*: *Enterococcus faecalis*
*P. mirabilis*: *Proteus mirabilis*
CFU: colony forming unit
ESR: erythrocyte sedimentation rate
MBD: minimal bactericidal dilution
TSP: 3-(trimethylsilyl)-propionic-2,2,3,3-d4 acid
NMR: nuclear magnetic resonance
MSUD: maple syrup urine disease
TNF: tumor necrosis factor
IFN: interferon
IL: interleukin
TGF: transforming growth factor
MMPs: matrix metalloproteinases
