# Supplementary figures and images for: Infected chronic ischemic wound topically treated with a multi-strain probiotic formulation: a novel tailored treatment strategy
Source: J Transl Med. 2019 Nov 9;17:364. doi: 10.1186/s12967-019-2111-0 (PMC6842486; doi:10.1186/s12967-019-2111-0)

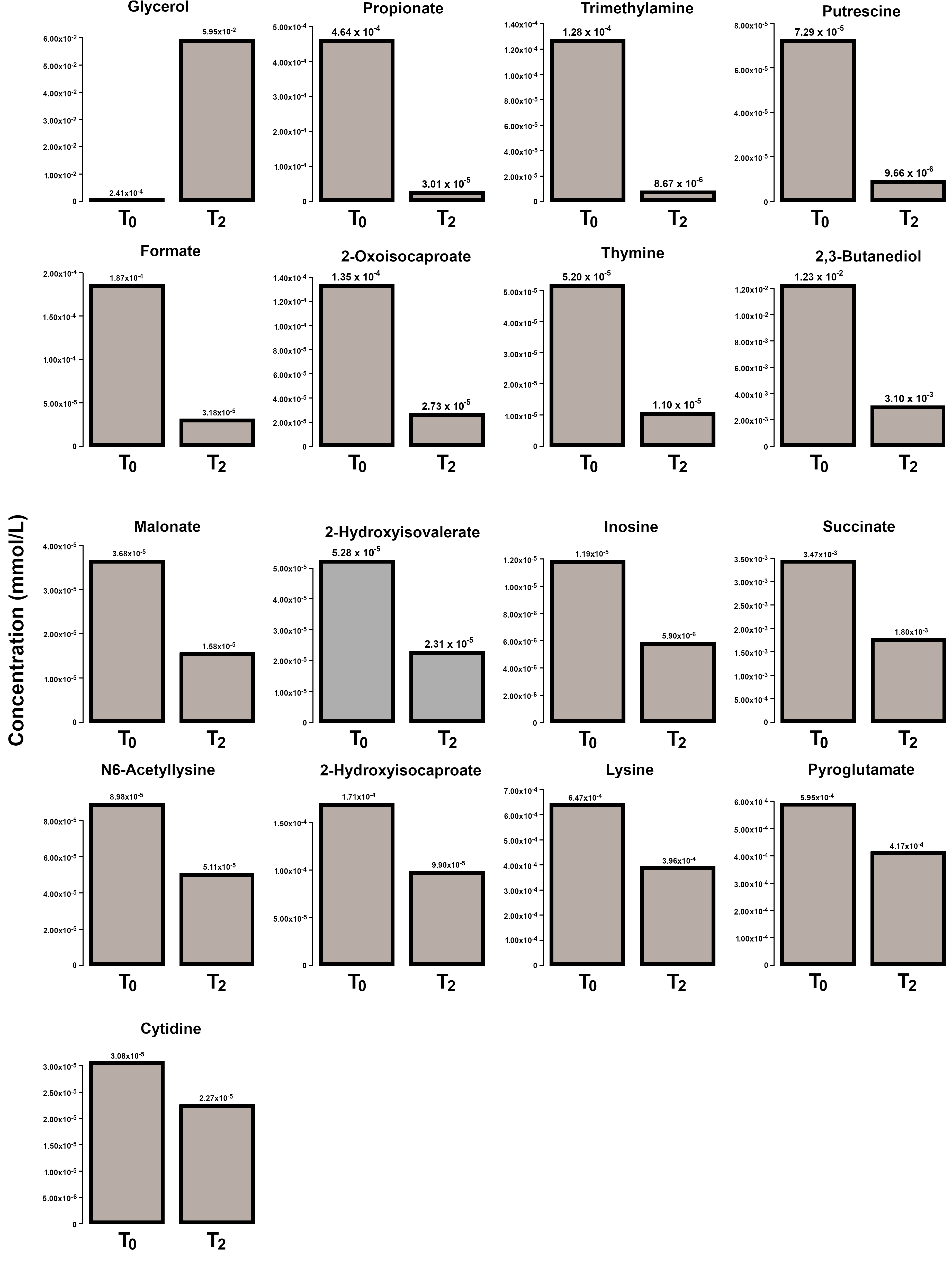

Supplement: Supplementary file 1 — Additional file 1: Figure S1. Molecules which concentration in the wound exudate was found to differ by more than 30% in connection to the treatment. [file 12967_2019_2111_MOESM1_ESM.tiff]
